# Cerebrospinal Fluid Biomarkers in Cerebral Amyloid Angiopathy: New Data and Quantitative Meta-Analysis

**DOI:** 10.3389/fnagi.2022.783996

**Published:** 2022-02-14

**Authors:** Nils G. Margraf, Ulf Jensen-Kondering, Caroline Weiler, Frank Leypoldt, Walter Maetzler, Sarah Philippen, Thorsten Bartsch, Charlotte Flüh, Christoph Röcken, Bettina Möller, Georg Royl, Alexander Neumann, Norbert Brüggemann, Benjamin Roeben, Claudia Schulte, Benjamin Bender, Daniela Berg, Gregor Kuhlenbäumer

**Affiliations:** ^1^Department of Neurology, University Medical Center Schleswig-Holstein, Campus Kiel, Kiel University, Kiel, Germany; ^2^Department of Radiology and Neuroradiology, University Medical Center Schleswig-Holstein, Campus Kiel, Kiel University, Kiel, Germany; ^3^Department of Neuroradiology, University Medical Center Schleswig-Holstein, Campus Lübeck, Lübeck, Germany; ^4^Institute of Clinical Chemistry, University Medical Center Schleswig-Holstein, Kiel/Lübeck, Germany; ^5^Department of Neurosurgery, University Medical Center Schleswig-Holstein, Campus Kiel, Kiel University, Kiel, Germany; ^6^Department of Pathology, University Medical Center Schleswig-Holstein, Campus Kiel, Kiel University, Kiel, Germany; ^7^Department of Neurology, University Medical Center Schleswig Holstein, Campus Lübeck, Lübeck, Germany; ^8^Institute of Neurogenetics, University of Lübeck, Lübeck, Germany; ^9^Department of Neurodegeneration, Hertie Institute for Clinical Brain Research, University of Tübingen, Tübingen, Germany; ^10^German Center for Neurodegenerative Diseases, University of Tübingen, Tübingen, Germany; ^11^Department of Neuroradiology, Diagnostical and Interventional Neuroradiology, University Hospital of Tübingen, Tübingen, Germany

**Keywords:** cerebral amyloid angiopathy (CAA), cerebrospinal fluid (CSF), high-precision electro-chemiluminescence immunoassay (ECLIA), Boston criteria, Alzheimer’s dementia (AD)

## Abstract

**Background:**

To evaluate the diagnostic accuracy of cerebrospinal fluid (CSF) biomarkers in patients with probable cerebral amyloid angiopathy (CAA) according to the modified Boston criteria in a retrospective multicentric cohort.

**Methods:**

Beta-amyloid 1-40 (Aβ40), beta-amyloid 1-42 (Aβ42), total tau (t-tau), and phosphorylated tau 181 (p-tau^181^) were measured in 31 patients with probable CAA, 28 patients with Alzheimer’s disease (AD), and 30 controls. Receiver-operating characteristics (ROC) analyses were performed for the measured parameters as well as the Aβ42/40 ratio to estimate diagnostic parameters. A meta-analysis of all amenable published studies was conducted.

**Results:**

In our data Aβ42/40 (AUC 0.88) discriminated best between CAA and controls while Aβ40 did not perform well (AUC 0.63). Differentiating between CAA and AD, p-tau^181^ (AUC 0.75) discriminated best in this study while Aβ40 (AUC 0.58) and Aβ42 (AUC 0.54) provided no discrimination. In the meta-analysis, Aβ42/40 (AUC 0.90) showed the best discrimination between CAA and controls followed by t-tau (AUC 0.79), Aβ40 (AUC 0.76), and p-tau^181^ (AUC 0.71). P-tau^181^ (AUC 0.76), Aβ40 (AUC 0.73), and t-tau (AUC 0.71) differentiated comparably between AD and CAA while Aβ42 (AUC 0.54) did not. In agreement with studies examining AD biomarkers, Aβ42/40 discriminated excellently between AD and controls (AUC 0.92–0.96) in this study as well as the meta-analysis.

**Conclusion:**

The analyzed parameters differentiate between controls and CAA with clinically useful accuracy (AUC > ∼0.85) but not between CAA and AD. Since there is a neuropathological, clinical and diagnostic continuum between CAA and AD, other diagnostic markers, e.g., novel CSF biomarkers or other parameters might be more successful.

## Introduction

Cerebral amyloid angiopathy (CAA) is the most frequent cause of lobar hemorrhage in the elderly. Its diagnosis predicts future hemorrhagic and ischemic complications (Yamada, 2000; Viswanathan and Steven, 2008; Wilson and Werring, 2017; Puy and Cordonnier, 2019). CAA is diagnosed using the modified Boston criteria (Linn et al., 2010). In clinical practice, the diagnosis of CAA is largely based on neuroradiological criteria because brain biopsies are rarely performed.

The direct measurement of amyloid proteins and other protein markers in cerebrospinal fluid (CSF) has been addressed in patients with sporadic CAA suggesting that measurement of beta-amyloid 1-40 (Aβ40), beta-amyloid 1-42 (Aβ42), total tau (t-tau), and phosphorylated tau 181 (p-tau^181^) in CSF might differentiate CAA from controls as well as from Alzheimer’s disease (AD) patients (Verbeek et al., 2009; Hernandez-Guillamon et al., 2012; Renard et al., 2012, 2016; Li et al., 2015; Martínez-Lizana et al., 2015; Charidimou et al., 2018; Banerjee et al., 2020). The Aβ42/40 discriminates excellently between AD and controls and changes of the Aβ40/42 ratio have been suggested to play a role in animal models of CAA (Janelidze et al., 2016; Gervaise-Henry et al., 2017; Doecke et al., 2020).

Beta-amyloid 1-40 more than Aβ42 is the main driver of beta-amyloid deposition in the wall of small cerebral arteries while Aβ42 is more important than Aβ40 in amyloid plaque formation in AD (Viswanathan and Greenberg, 2011). The results of previous diagnostic studies were inhomogeneous and a diagnostic algorithm including cut-off values is lacking. In particular, the difficult differentiation between CAA and AD patients might be caused by the overlapping pathomechanisms (Kester et al., 2014; Greenberg et al., 2020).

We aimed to determine the diagnostic value of CSF Aβ40, Aβ42, t-tau, and p-tau^181^ and the ratio Aβ42/40 (mathematically equivalent to: Aβ40/42) using an automated high-precision electro-chemiluminescence immunoassay (ECLIA) in a large sample of patients with probable CAA. The modified Boston criteria were used as the diagnostic standard for CAA. Also, we performed a quantitative meta-analysis of all amenable studies of CSF parameters in CAA. Diagnostic parameters in our samples, as well as the meta-analysis, were determined using receiver-operating characteristics (ROC) analysis.

## Materials and Methods

### Participants

We retrospectively included three groups between May 2014 and November 2019 at the University Medical Center Schleswig-Holstein (Kiel and Lübeck) and the University Hospital Tübingen: CAA, AD, and control participants.

#### Cerebral Amyloid Angiopathy Group

Inclusion criteria for the CAA group were (1) probable CAA or probable CAA with supporting pathology according to the modified Boston criteria (Table 1); (2) availability of a diagnostic cranial MRI including a gradient-echo T2* or susceptibility-based sequence (SWI, SWIp, or veno BOLD); and (3) availability of a CSF sample within 3 months before or after the cerebral MRI grafted for clinical purposes during the diagnostic work-up.

**TABLE 1:** Modified Boston criteria for the diagnosis of CAA.

Definite CAA	Full postmortem examination demonstrating:
	• Lobar, cortical, or corticosubcortical hemorrhage
	• Severe CAA with vasculopathy
	• Absence of other diagnostic lesion
Probable CAA with supporting pathology	Clinical data and pathologic tissue (evacuated hematoma or cortical biopsy) demonstrating:
	• Lobar, cortical, or corticosubcortical hemorrhage
	• Some degree of CAA in specimen
	• Absence of other diagnostic lesion
Probable CAA	Clinical data and MRI or CT demonstrating:
	• Multiple hemorrhages restricted to lobar, cortical, or corticosubcortical regions (cerebellar hemorrhage allowed)
	or
	• Single lobar, cortical, or corticosubcortical hemorrhage and focal or disseminated superficial siderosis
	• Age ≥ 55 years
	• Absence of other cause of hemorrhage or superficial siderosis
Possible CAA	Clinical data and MRI or CT demonstrating:
	• Single lobar, cortical, or corticosubcortical hemorrhage
	• Focal or disseminated superficial siderosis
	• Age ≥ 55 years
	• Absence of other cause of hemorrhage or superficial siderosis

Patients with possible CAA according to the modified Boston criteria (Table 1) or any genetically determined or inflammatory forms of CAA were excluded.

#### Alzheimer’s Disease Group

Inclusion criteria for the AD group were (1) fullfilment of the diagnostic criteria of Alzheimer’s dementia according to the National Institute on Aging and Alzheimer’s Association (NIA-AA) (Jack et al., 2011); (2) availability of a diagnostic cranial MRI including a gradient-echo T2* or susceptibility-based sequence (SWI, SWIp, or veno BOLD); and (3) availability of a CSF sample within 3 months before or after the cerebral MRI grafted for clinical purposes during the diagnostic work-up.

Exclusion criteria were imaging features of probable CAA according to the modified Boston criteria (Table 1).

#### Control Group

Controls were recruited at the University Medical Center Schleswig-Holstein (Kiel and Lübeck) and the University Hospital Tübingen with biomaterial from the Hertie Institute for Clinical Brain Research Biobank (Tübingen) or the UKSH biobank at the Institute of Clinical Chemistry.

Inclusion criteria for the control group were (1) complaints suggestive of neurologic disease, but no evidence of organic central nervous system disease was found after thorough diagnostic work-up and (2) availability of a CSF sample within 3 months before or after the occurrence of neurological complaints.

Exclusion criteria were the evidence or a history of a disease of the central nervous system including abnormal routine CSF parameters or if available, relevant pathological findings in a cranial MRI.

We excluded patients from all groups with a competing central nervous system disease that might increase the level of any of the analyzed CSF parameters. Patients with cerebral infarcts >1.5 cm in diameter or an intracerebral hemorrhage within 4 months before the lumbar puncture were excluded to rule out elevated values for the axonal damage marker t-tau (Hjalmarsson et al., 2014). We only made an exception for CAA patients whose surgical treatment of hemorrhages resulted in a pathological verification of CAA (*n* = 5). In these cases, we confirmed that the CSF t-tau concentrations were in the typical range of the other CAA patients. Further, patients who had a history of heart-lung-machine (HLM) procedures were also excluded because HLM treatment can mimic CAA on MRI scans (Jeon et al., 2010).

All MRI scans including the radiological classification of the CAA cases were reviewed and rated by a board-certified neuroradiologist (UJ-K) blinded to history, clinical diagnosis and laboratory parameters. Imaging parameters were rated based on the STRIVE criteria (Wardlaw et al., 2013).

Furthermore, we matched the three groups on a group level according to sex and age.

### Clinical Data

We extracted clinical data (age, sex, date and reason of hospital admission, pre-existing diseases, medication and blood coagulation parameters on admission, risk factors for vascular diseases, date and reason of MRI and lumbar puncture, and results of cognitive assessments) from the medical records.

### Cerebrospinal Fluid Analysis

Cerebrospinal fluid was stored at −80° in participating biobanks following lumbar puncture. All participating biobanks used polypropylene primary tubes and samples were frozen at −80°C in polypropylene secondary tubes within maximal 48 h at 4°C. Samples were retrieved for this study and kept on dry ice until the time of measurements. Samples were thawed and aliquoted for measurements into certified polypropylene tubes immediately before measurement. Analysis was performed on a fully automated platform (FujiRebio LumiPulse) using dedicated ECLIA assay chemistry (FujiRebio©) according to the manufacturer’s protocols including calibration and controls. All analytes were measured within 2 days using single measurements. Predetermined coefficients of variations (CV) for this approach showed intra- and inter-assay variations of <4% for Aβ40, Aβ42, p-Tau, and 7% for t-tau across the whole measurement range. The reader, a board certified laboratory physician (FL), extracting the data was blinded to history, clinical diagnosis and other laboratory parameters of the study participants. The assay is approved and in routine clinical use at the University Hospital Schleswig-Holstein, Kiel.

### Statistics

We used R version 3.6.3 for all analyses. We assessed age and biomarker concentration differences between the three study groups (CAA, AD, and controls) using analysis-of-variance (ANOVA) and *post hoc* Tukey test if applicable. To analyze the sex distribution between groups we used Fisher’s exact test and for differences in biomarker concentrations between sexes the unpaired *t*-tests per group. The correlation between CSF storage time and biomarker concentrations was analyzed using Kendall rank correlation analysis. To assess the diagnostic value of the CSF biomarkers we used ROC-analysis in pairwise comparisons between the diagnostic groups. ROC analyses were performed using the pROC-package (version 1.16.2) including calculation of the area under the curve (AUC) and its confidence intervals as well as sensitivity, specificity, and cut-off values optimized using the Youden index. We compared the AUCs between biomarkers using the function “roc.test” from the pROC package for statistically significant differences. The statistical significance threshold was *p* < 0.05.

The Ethics Committee of the Medical Faculty of the University of Kiel, Lübeck and Tübingen approved this retrospective study (B 255/18, AZ19-108, and 864/2016BO2). The study was conducted following the World Medical Association Declaration of Helsinki. Anonymized data will be shared by request with any qualified investigator.

### Study Selection and Statistics of the Meta-Analysis

**FIGURE 1:**
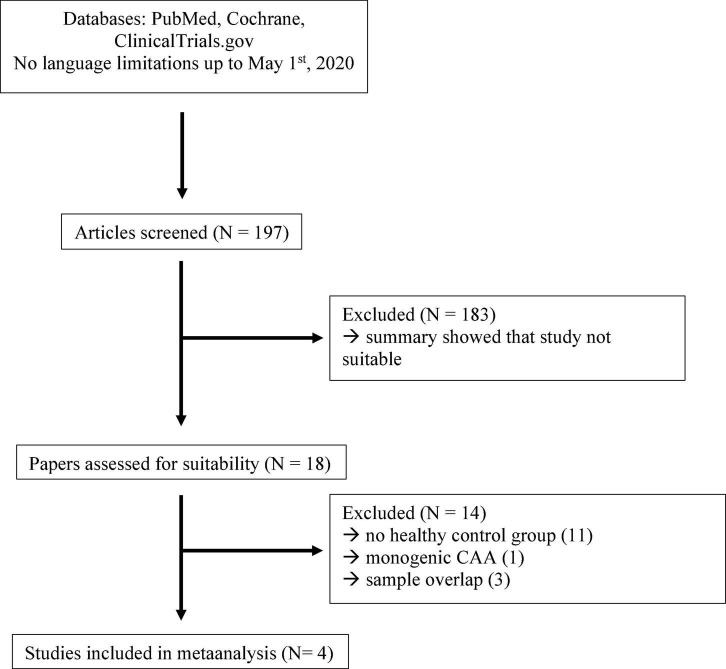
Flow chart of study selection for the meta-analysis.

We [two board certified neurologists (GK and NGM)] searched PubMed (up to May 1st, 2020) with the search term: (CAA OR cerebral amyloid angiopathy OR cerebral-amyloid-angiopathy) AND (cerebrospinal fluid OR CSF) and in addition, the Cochrane database^1^ and the clinical trials database^2^. We found no studies in the Cochrane or Clinical Trials database. From 197 entries in PubMed, we selected 18 studies, which had done analysis of at least one of the following four biomarkers: Aβ40, Aβ42, t-tau, and p-tau^181^. Next, we checked whether studies fulfilled the following four hierarchical inclusion criteria: (1) sporadic CAA diagnosed according to the original or modified Boston criteria, (2) a control group, either healthy or suffering from conditions without known influence on biomarker levels, (3) measurement of Aβ40, Aβ42, t-tau, and p-tau^181^ in CSF, and (4) individual biomarker data extractable from figures or tables. The following reasons for exclusion were agreed on in consensus: studies on animals, studies on CAA-related inflammation, case reports and letters to the editor. The flow chart (Figure 1) illustrates the selection process according to the PRISMA criteria. If more than one reason for exclusion of a study was present, only the most important reasons for exclusion were given in Figure 1. Four studies showed a strong sample overlap and we used only one of them for the meta-analysis. We extracted scatterplots with individual level CSF concentrations of the biomarkers from the included publications and determined individual level biomarker concentrations with the software ‘‘PlotDigitizer’’^3^ as previously described (Jelicic Kadic et al., 2016). We used frequency histograms to examine the distribution of biomarker concentrations in the control group for compatibility with a normal distribution. Per study, we calculated the mean and SD of each biomarker in the control group. *Z*-scores were calculated for every sample in each study per biomarker by subtracting the mean of the control group from each value in the study and dividing the result by the SD of the control group. We performed ROC analysis on the *Z*-scores as described. Subsequently, we report the results of the meta-analysis including all retrieved studies without our study (WO) and including our study (ALL).

## Results

### Clinical and MRI Data

**FIGURE 2:**
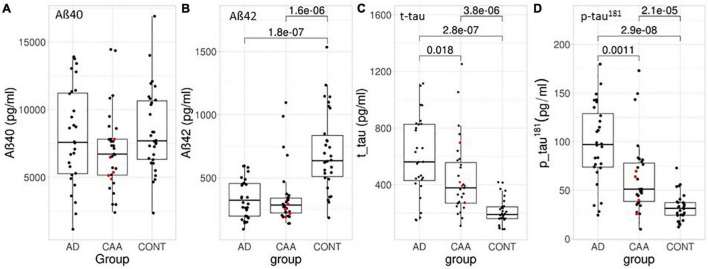
Box- and scatterplots of the CSF biomarker values in this study per diagnostic group. (A) Aβ40, (B) Aβ42, (C) t-tau, and (D) p-tau^181^. Red dots indicate the pathologically confirmed CAA samples. All concentrations in pg/ml. The box encompasses 50% of the samples and the whiskers extend 1.5 quartiles to each side of the box. Significant differences are indicated including *p*-values.

In the CAA group we included 31 patients, including 26 patients with probable CAA and 5 patients with probable CAA with supporting pathology according to the modified Boston criteria. A total of 13 patients presented with pathologies on cranial MRI (intracranial hemorrhage *n* = 3, subarachnoid hemorrhage *n* = 3, acute infarct <1.5 cm *n* = 7). In these cases, we confirmed that the CSF t-tau concentrations were in the typical range of the other CAA patients (as indicated by red dots in Figure 2). A total of 13 patients were categorized as “demented” and 7 patients as having a mild cognitive impairment based on a neuropsychological screening test as performed during hospital stay [Mini Mental Status Examination, Montreal Cognitive Assessment, Mattis Dementia Rating Scale or DemTect (Kalbe et al., 2004)]. Four patients showed no cognitive deficits and no information was available in seven patients.

In the AD group we included 28 patients. Two of the AD patients (both with one microbleed each, in cerebellar and periventricular location, respectively, not compatible with possible or probable CAA) exhibited cerebral microbleeds.

In the control group we included 30 patients. A total of 12 patients presented with complaints suggestive of neurologic disease, but no evidence of organic neurologic disease was found after thorough diagnostic work-up, 9 patients had a peripheral neurological disease, 2 patients had a muscle disease, and 7 participants were healthy controls without a neurological disease from the biobank of the Hertie Institute, Tübingen. A total of 14 patients (47%) had a cerebral MRI available for review. A neuropsychological screening available in seven patients (23%) showed no indication of dementia.

Table 2 summarizes the main characteristics of the three groups. Age (*p* = 0.08, ANOVA) and gender distribution (*p* = 0.4, Chi square) did not differ significant between the three groups. Most of the MRIs were performed at 1.5 T (79%), however, that was consistent between the three groups (*p* = 0.9, Chi square). A majority (59%) of MRIs included a susceptibility-based sequence instead of a GRE T2* with significantly more susceptibility-based sequences performed in the CAA group than in the other groups (87 vs. 36 vs. 43%, *p* = 0.00002, Fischer’s exact test).

**TABLE 2:** Clinical and MRI patients characteristics.

		CAA (*n* = 31)	AD (*n* = 28)	Controls (*n* = 30)
Center (*n*/%)		Kiel: 22/71	Kiel: 13/47	Kiel: 17/57
		Lübeck: 4/13	Lübeck: 4/14	Lübeck: 4/13
		Tübingen: 5/16	Tübingen: 11/39	Tübingen: 9/30
Gender (female) (*n*/%)		13/42%	16/57	17/56
Age years [mean (SD)]		75.1 (5.3)	71.1 (7.7)	72.5 (7.8)
MRI field strength (*n*/%)	1.5 Tesla	25/81	22/79	11/79
	3 Tesla	6/19	6/21	3/21
Sequence (*n*/%)	Susceptibility based	27/87	10/36	6/43
	T2* GRE	4/13	18/64	0/0
ICH (*n*/%)	Acute	3/10	0/0	0/0
	Chronic	5/16	0/0	0/0
cSS (*n*/%)		14/45	0/0	0/0
SAH (*n*/%)		3/10	0/0	0/0
Acute infarct^+^ (*n*/%)	Embolic	3/10	0/0	0/0
	Lacune	4/13	0/0	0/0
Chronic infarct (*n*/%)	Embolic	3/10	3/11	1^§^/3
	Lacune	7/23	2/7	0/0

*AD, Alzheimer’s disease; CAA, cerebral amyloid angiopathy; cSS, cortical superficial siderosis; GRE, gradient-echo; ICH, intracranial hemorrhage; N/A, not available; SAH, subarachnoid hemorrhage.*

*^+^Cerebral infarcts <1.5 cm in diameter.*

*^§^ Asymptomatic, diagnosed on MRI.*

### Cerebrospinal Fluid Data

We analyzed the CSF parameters Aβ40, Aβ42, t-tau, and p-tau^181^ and the ratio Aβ42/40 which is mathematically equivalent to the multiplicative inverse Aβ40/42. Table 3 and Figure 2 show a comparison of biomarker concentrations between groups. Aβ42 was decreased in both the AD and CAA group when compared to the control group but there was no significant difference between CAA and AD. Aβ40 did not differ significantly between CAA, AD, and controls. T-tau and p-tau^181^ were highest in the AD group followed by the CAA group and controls and all group differences were significant (Figure 2).

**TABLE 3:** Cerebrospinal fluid parameters of the samples in our study.

Parameter	CAA *n* = 31 Mean (SD)	AD *n* = 28 Mean (SD)	CONT *n* = 30 Mean (SD)	ANOVA *p*-value
Aβ40	7008 (2896)	7997 (3649)	8443 (3102)	0.21
Aβ42	347 (228)	340 (154)	709 (317)	<0.01
T-tau	444 (259)	597 (280)	211 (91)	<0.01
P-tau^181^	62 (37)	98 (41)	32 (14)	<0.01

*AD, Alzheimer’s disease; CAA, cerebral amyloid angiopathy; CONT, controls; ANOVA across all three groups. Significant differences between pairs are indicated in Figure 2.*

Most CSF biomarker concentrations in samples of patients with probable CAA with supporting pathology were within the interquartile range and the remaining ones were located within the range of ±1.5 quartiles of all values (indicated by red dots in Figure 2). The same applied to patients with other acute radiological findings as described above and indicated in Table 2.

**FIGURE 3:**
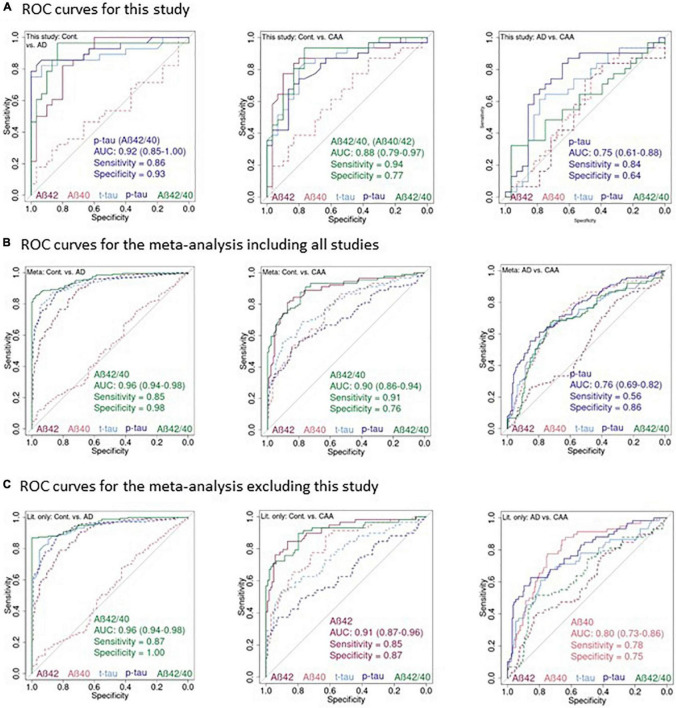
Receiver-operating characteristics curves for the CSF parameters in our study and the meta-analyses. **(A)** ROC plots of Aβ40, Aβ42, t-tau, p-tau^181^, and Aβ42/40 in this study for: controls vs. AD, controls vs. CAA, AD vs. CAA. **(B)** The same plots as in **(A)** for the meta-analysis including this study. **(C)** And excluding this study. CSF parameters corresponding to the lines are color-coded at the bottom of the plots. Solid lines: parameter with the highest AUC and parameters with an AUC not significantly different from the highest AUC. Dashed line: parameters with a significantly smaller AUC compared to the one with the highest AUC. Text insert: AUC, 95% confidence interval of the AUC, sensitivity, specificity of the parameter with the highest AUC and, if applicable name of additional parameter with identical AUC.

Cerebrospinal fluid-storage time did not correlate with biomarker concentrations (Aβ40 *p* = 0.21, Aβ42 *p* = 0.42, t-tau *p* = 0.08, p-tau^181^ *p* = 0.12) and biomarker concentrations did not differ significantly between sexes within a diagnostic group (*p*-value range: 0.09–0.98). Figure 3A and Table 4 show the ROC curves, the key diagnostic parameters, AUC with 95% confidence interval, sensitivity, and specificity.

**TABLE 4:** Diagnostic parameters determined in this study and the meta-analyses.

This study	Parameter	AUC (95% CI)	Sensitivity (95% CI)	Specificity (95% CI)	*Z*-score cut-off	Measured cut-off
cont/ad	Aβ40	0.53 (0.38–0.69)	0.32 (0.14–0.5)	0.83 (0.7–0.97)	–0.21	5904
cont/ad	Aβ42	0.87 (0.78–0.96)	0.82 (0.68–0.96)	0.8 (0.67–0.93)	–0.2	490
**cont/ad**	**P-tau^181^**	**0.92 (0.85–1)**	**0.86 (0.71–0.96)**	**0.93 (0.83–1)**	**0.38**	**55**
cont/ad	T-tau	0.89 (0.8–0.99)	0.82 (0.68–0.93)	0.93 (0.83–1)	0.92	381
**cont/ad**	**A**β**42/40**	**0.92 (0.83–1)**	**0.96 (0.89–1)**	**0.83 (0.7–0.93)**	**−−1.22**	**0.06**
cont/caa	Aβ40	0.63 (0.49–0.77)	0.77 (0.61–0.9)	0.47 (0.3–0.63)	0.99	7950
cont/caa	Aβ42	0.86 (0.76–0.96)	0.77 (0.61–0.9)	0.9 (0.77–1)	–0.71	347
cont/caa	P-tau^181^	0.82 (0.71–0.93)	0.68 (0.52–0.84)	0.87 (0.73–0.97)	–0.36	45
cont/caa	T-tau	0.85 (0.75–0.94)	0.81 (0.65–0.94)	0.8 (0.67–0.93)	–0.1	258
**cont/caa**	**A**β**42/40**	**0.88 (0.79–0.97)**	**0.94 (0.84–1)**	**0.77 (0.6–0.9)**	**−−0.58**	**0.07**
ad/caa	Aβ40	0.58 (0.43–0.73)	0.81 (0.68–0.94)	0.46 (0.29–0.64)	1.38	8618
ad/caa	Aβ42	0.54 (0.39–0.7)	0.74 (0.58–0.9)	0.5 (0.32–0.68)	–0.75	338
**ad/caa**	**P-tau^181^**	**0.75 (0.61–0.88)**	**0.84 (0.71–0.94)**	**0.64 (0.46–0.82)**	**2.35**	**83**
ad/caa	T-tau	0.68 (0.54–0.82)	0.61 (0.45–0.77)	0.79 (0.61–0.93)	1.13	406
ad/caa	Aβ42/40	0.61 (0.46–0.75)	0.32 (0.16–0.48)	0.96 (0.89–1)	–1.4	0.05

**Meta-analysis excluding this study**	**Parameter**	**AUC (95% CI)**	**Sensitivity (95% CI)**	**Specificity (95% CI)**	***Z*-score cut-off**	**Measured cut-off**

cont/ad	Aβ40	0.53 (0.46–0.6)	0.68 (0.61–0.74)	0.43 (0.33–0.52)	0.19	N/A
cont/ad	Aβ42	0.89 (0.85–0.93)	0.79 (0.73–0.85)	0.83 (0.75–0.89)	–0.96	N/A
cont/ad	P-tau^181^	0.93 (0.9–0.96)	0.89 (0.84–0.93)	0.84 (0.76–0.9)	0.78	N/A
cont/ad	T-tau	0.94 (0.91–0.96)	0.86 (0.81–0.91)	0.89 (0.84–0.95)	1.04	N/A
**cont/ad**	**A**β**42/40**	**0.96 (0.94–0.98)**	**0.87 (0.82–0.91)**	**1 (1–1)**	**−−1.93**	**N/A**
cont/caa	Aβ40	0.82 (0.75–0.89)	0.88 (0.79–0.95)	0.63 (0.53–0.72)	–0.42	N/A
**cont/caa**	**A**β**42**	**0.91 (0.87–0.96)**	**0.85 (0.75–0.93)**	**0.87 (0.8–0.93)**	**−−1.17**	**N/A**
cont/caa	P-tau^181^	0.65 (0.56–0.74)	0.37 (0.25–0.49)	0.92 (0.88–0.97)	1.47	N/A
cont/caa	T-tau	0.76 (0.68–0.84)	0.53 (0.39–0.66)	0.91 (0.86–0.96)	1.31	N/A
cont/caa	Aβ42/40	0.9 (0.85–0.96)	0.9 (0.81–0.97)	0.8 (0.71–0.87)	–0.83	N/A
**ad/caa**	**A**β**40**	**0.8 (0.73–0.86)**	**0.78 (0.67–0.88)**	**0.75 (0.68–0.81)**	**−−0.73**	**N/A**
ad/caa	Aβ42	0.6 (0.51–0.68)	0.39 (0.27–0.53)	0.84 (0.78–0.89)	–2.14	N/A
ad/caa	P-tau^181^	0.78 (0.71–0.86)	0.63 (0.51–0.75)	0.85 (0.8–0.91)	1.06	N/A
ad/caa	T-tau	0.72 (0.64–0.8)	0.68 (0.56–0.8)	0.73 (0.67–0.79)	2.07	N/A
ad/caa	Aβ42/40	0.65 (0.56–0.73)	0.52 (0.4–0.64)	0.82 (0.77–0.88)	–2.95	N/A

**Meta-analysis including this study**	**Parameter**	**AUC (95% CI)**	**Sensitivity (95% CI)**	**Specificity (95% CI)**	***Z*-score cut-off**	**Measured cut-off**

cont/ad	Aβ40	0.53 (0.47–0.59)	0.75 (0.7–0.81)	0.33 (0.24–0.41)	0.46	N/A
cont/ad	Aβ42	0.88 (0.85–0.92)	0.88 (0.84–0.93)	0.72 (0.63–0.79)	–0.6	N/A
cont/ad	P-tau^181^	0.93 (0.9–0.95)	0.88 (0.84–0.92)	0.84 (0.78–0.9)	0.78	N/A
cont/ad	T-tau	0.93 (0.9–0.96)	0.88 (0.83–0.92)	0.87 (0.81–0.92)	0.96	N/A
**cont/ad**	**A**β**42/40**	**0.96 (0.94–0.98)**	**0.85 (0.8–0.9)**	**0.98 (0.94–1)**	**−−1.74**	**N/A**
cont/caa	Aβ40	0.76 (0.69–0.82)	0.87 (0.8–0.93)	0.54 (0.44–0.63)	–0.2	N/A
cont/caa	Aβ42	0.89 (0.84–0.94)	0.81 (0.73–0.89)	0.87 (0.81–0.93)	–1.17	N/A
cont/caa	P-tau^181^	0.71 (0.63–0.78)	0.52 (0.42–0.62)	0.84 (0.78–0.9)	0.86	N/A
cont/caa	T-tau	0.79 (0.73–0.85)	0.69 (0.6–0.78)	0.78 (0.71–0.85)	0.59	N/A
**cont/caa**	**A**β**42/40**	**0.9 (0.86–0.94)**	**0.91 (0.84–0.97)**	**0.76 (0.68–0.83)**	**−−0.73**	**N/A**
ad/caa	Aβ40	0.73 (0.67–0.79)	0.74 (0.65–0.83)	0.65 (0.58–0.71)	–0.48	N/A
ad/caa	Aβ42	0.54 (0.47–0.61)	0.81 (0.72–0.89)	0.32 (0.25–0.38)	–1.16	N/A
ad/caa	**P-tau^181^**	**0.76 (0.69–0.82)**	**0.56 (0.46–0.66)**	**0.86 (0.8–0.9)**	**1.06**	**N/A**
ad/caa	T-tau	0.71 (0.64–0.77)	0.66 (0.56–0.76)	0.73 (0.67–0.79)	2.11	N/A
ad/caa	Aβ42/40	0.69 (0.63–0.76)	0.67 (0.57–0.76)	0.73 (0.67–0.78)	–2.86	N/A

*CONT, controls; AD, Alzheimer’s disease; CAA, cerebral amyloid angiopathy; AUC, area under the curve in the ROC analysis; Z-score cut-off, Z-score used as cut-off to determine optimal sensitivity and specificity in the metaanalysis. Measured cut-off: cut-off of directly measured parameters to determine optimal sensitivity and specificity in this stud. N/A, not applicable. Bold: parameter with the highest AUC.*

For the comparison between controls vs. AD we observed the largest AUCs of 0.92 for p-tau^181^ and Aβ42/40, for the comparison controls vs. CAA we observed the largest AUC of 0.88 for Aβ42/40, and for the comparison AD vs. CAA we observed the largest AUC of 0.75 for p-tau^181^.

For the comparison between controls with AD and CAA patients, respectively, the AUC did not differ significantly between all markers except Aβ40 which showed a significantly smaller AUC (Table 4 and Figure 3A). The same was true for the comparison between AD and CAA patients for p-tau^181^, t-tau, and Aβ42/40 while Aβ40 and Aβ42 performed significantly worse.

The Youden index optimized measures of sensitivity and specificity (Table 4), should be interpreted with caution. ROC curves are jagged due to the relatively small sample size and some ROC curves (e.g., Aβ42 for Alzheimer vs. CAA) run for long stretches nearly parallel to the bisecting line. This indicates that several sensitivity/specificity combinations would result in nearly equal Youden indices.

### Meta-Analysis

We identified five studies including this study suitable for a meta-analysis based on individual CSF biomarker concentrations (Figure 1). Table 5 gives an overview of these studies incorporating in total 90 CAA patients, 204 AD patients, and 134 controls. We used *Z*-scores to harmonize the biomarker data generated on different laboratory platforms between studies. Subsequently, we performed the same ROC analyses as for the data in this study. Table 4 provides the key diagnostic parameter data for this study as well as the meta-analyses including absolute cut-off values for our study and optimal cut-off *Z*-values for the meta-analysis. Figure 3B shows the ROC plots for the meta-analysis of all studies (ALL) and Figure 3C for the meta-analysis without this study (WO). The differentiation between controls and AD was excellent in both meta-analyses with an AUC of 0.96 for the Aβ42/40 ratio in the ALL and the WO analysis. Aβ42 (AUC 0.88 ALL, 0.89 WO) was significantly worse and Aβ40 (AUC 0.53 ALL and WO) did not provide any differentiation at all. Aβ42/40 (AUC 0.9 for ALL and WO) and Aβ42 (AUC 0.89 ALL, 0.91 WO) alone showed comparable AUCs in the analysis of controls vs. CAA. T-tau, Aβ40, and p-tau^181^ performed worse. P-tau (AUC 0.76 ALL, 0.78 WO), Aβ40 (AUC 0.73 ALL, 0.80 WO), and t-tau (AUC 0.71 ALL, 0.72 WO) showed comparable differentiation between AD and CAA while Aβ42 (AUC 0.54 ALL, 0.60 WO) did not separate these diagnoses.

**TABLE 5:** Characteristics of the studies in the meta-analysis.

Study	Group demographics (*n*, age, and cognitive status)	Diagnostic criteria CAA (MRI sequence), AD	Method of CSF analysis	Results
Verbeek et al., 2009	– **CAA:** *n* = 17, age 62.8 ± 11.9 years (mean, SD), not demented – **AD:** *n* = 72, age 69.4 ± 8.3 years (mean, SD) – **Controls:** *n* = 58, age 61.0, ± 8.7 years (mean, SD), no neurological disorders	CAA: probable or definite per Boston criteria (T2*) AD: NINCDS-ADRDA	ELISA (Innogenetics, NV, Gent, Belgium)	• Aβ40 and Aβ42 decreased in CAA vs. AD and controls • T-tau and p-tau^181^ increased in CAA vs. controls but decreased vs. AD
Martínez-Lizana et al., 2015	– **CAA:** *n* = 19 [12 CAA without SAH, age 69.8 years (mean), 7 CAA with SAH, age 79.1 years (mean)], predominantly MCI/dementia – **AD:** *n* = 42, age 67.6, 50.6–79.8 (mean, range) – **Controls:** *n* = 20, age 66.5, 55.7–77.5 (mean, range), no cognitive complaints, normal neuropsychological evaluation	CAA: possible or probable per modified Boston criteria (T2*) AD: NINCDS-ADRDA	ELISA (Innogenetics, NV, Gent, Belgium)	• Aβ40 and Aβ42 decreased in CAA vs. controls but not AD • T-tau increased in CAA vs. controls • T-tau and p-tau^181^ increased in AD vs. CAA
Renard et al., 2016	– **CAA:** *n* = 13, age 73 years (median), no pre-existing cognitive deficits as reported by patient or family – **AD:** *n* = 42, age 73 years (median) – **Controls:** *n* = 16, age 70 years (median), no neurological diseases related to amyloid deposition, no healthy controls	CAA: possible or probable per Boston criteria (T2*, optional SWI) AD: NIA-AA	ELISA (Innogenetics, NV, Gent, Belgium)	• Aβ42 decreased in CAA vs. controls but not AD • Aβ40 CAA decreased in CAA vs. AD but not controls • T-tau decreased in CAA vs. AD, decreased in CAA vs. controls • P-tau^181^ increased in CAA vs. AD but not controls
Banerjee et al., 2020	– **CAA:** *n* = 10, age 68.6 ± 3.0 years (mean, SD), MMSE ≥ 23 – **AD:** *n* = 20, age 62.5 ± 4.1 years (mean, SD) – **Controls:** *n* = 10, age 62.2 ± 5.4 years (mean, SD), no significant neurological disease	CAA: probable per modified Boston criteria AD: amnestic symptoms, CSF criteria	ECL, Meso Scale Discovery V-PLEX Aβ peptide panel 1; ELISA (Innotest, Fujirebio Europe, Gent, Belgium)	• Aβ40 and Aβ42 decreased in CAA vs. AD and controls • P-tau^181^ and p-tau^181^ increased in AD vs. CAA and controls, but not CAA vs. controls
Our study	– **CAA:** *n* = 31, age 75.1 ± 5.3 years (mean, SD), 54% demented – **AD:** *n* = 28, age 71.1 ± 7.7 years (mean, SD) – **Controls:** *n* = 30, age 72.5 ± 7.8 years (mean, SD) 1/4 healthy controls, 1/3 peripheral neurological diseases	CAA: probable or probable with supporting pathology per modified Boston criteria (SWI, T2*) AD: NIA-AA	Lumipulse 2. Gen. FujiRebio	• Aβ42 decreased in CAA vs. controls and AD vs. controls but not CAA vs. AD • Aβ40 not different in CAA, AD, and controls • T-tau and p-tau^181^ decreased in CAA vs. AD group, and CAA vs. controls and AD vs. controls

*AD, Alzheimer’s disease; CAA, cerebral amyloid angiopathy; SAH, atraumatic convexal subarachnoid hemorrhage; CSF, cerebrospinal fluid; ECL, electrochemiluminescence; ELISA, enzyme-linked immunosorbent assay; MCI, mild cognitive impairment; NIA-AA, National Institute on Aging and Alzheimer’s Association; NINCDS-ADRDA, National Institute of Neurological and Communicative Disorders and Stroke and the Alzheimer’s Disease and Related Disorders Association; SWI, susceptibility-weighted imaging.*

## Discussion

Taken together, the results of this study and the meta-analyses indicate that CSF Aβ42 and the Aβ42/40 ratio separates controls from CAA patients with good accuracy (AUC 0.86–0.91). However, the differentiation between AD and CAA on the basis of CSF standard parameters proves to be more difficult. Our quantitative meta-analysis of all amenable CAA studies on CSF parameters utilizing control groups shows that Aβ40, t-tau, and p-tau^181^ yield very similar diagnostic results for AD vs. CAA with an AUC in the range of 0.71–0.80. In our opinion, these values are insufficient to justify routine clinical use.

In contrast to the meta-analysis, Aβ40 did not differentiate between AD and CAA (AUC 0.58) in our study. Especially the size of the actual CAA group is larger than in all preceding studies investigating this topic, and also the control group is larger than in almost all previous studies [except (Verbeek et al., 2009)]. Additional strengths are the use of highly accurate CSF analysis using an automated platform with minimal variation coefficient and strict adherence to diagnostic criteria. The main limitation of the study is its retrospective design. Also, complete and homogeneous neuropsychological test data would have been of great use to us, enabling us to correlate CSF biomarker concentrations not only with the diagnostic category but also with cognitive performance. We speculate that this could be one factor influencing the performance of Aβ40 as a biomarker for CAA (van Oijen et al., 2006). In contrast to the studies by Verbeek et al. (2009), Renard et al. (2012, 2016), and Banerjee et al. (2020), most CAA patients in our cohort were cognitively impaired. We included these patients because they fulfilled the modified Boston criteria that do not contain a statement concerning the cognitive status. We included patients over a long period of time (2014–2019) in three large tertiary care centers and thus believe that they depict a representative sample of CAA patients encountered in clinical practice. On the other hand, a large proportion of the CAA patients in the study by Martínez-Lizana et al. (2015) were also cognitively impaired and while the number of cases was low, results shown in their Figure 2 suggest that Aβ40 does not reliably differentiate between AD and CAA. Due to the small number of cases in our study a comparison between demented (*n* = 17) and non-demented (*n* = 4) CAA patients did not yield any statistically meaningful results.

Since the modified Boston criteria rely in large part on imaging criteria and microbleed detection, the choice of sequence is crucial. Susceptibility based sequences can detect substantially more microbleeds (Cheng et al., 2013). Due to the retrospective nature of the study we could not fully harmonize the use of image parameters and sequences across centers and we thus cannot fully rule out that this introduced a selection bias. However, the established diagnostic criteria for the diagnosis of CAA and AD, namely the modified Boston criteria and the NIA-AA were fulfilled for all subjects. A further source of error is the unknown relationship between CSF parameters and APOE4 allele status, which was not systematically captured in this study. Further, it has to be noted that CAA is considered an umbrella diagnosis with a spectrum of different manifestations concerning the presence of, e.g., atraumatic SAH, cortical siderosis, intracranial parenchymal hemorrhage, and clinical features such as cognitive impairment, presence of transient focal neurological episodes. It is unknown whether all these different manifestations share the same pathophysiological mechanism and degree of amyloid deposition and thus its concentration in CSF.

Absolute values of CSF biomarker measurements differ between different laboratories and using different methodologies. Therefore, they are not directly comparable even though all assay manufacturers use the same concentration units (pg/ml). To extract the maximum amount of data possible from published studies we re-digitized the published plots to get individual level biomarker concentrations. To homogenize measurements between different publications we generated *Z*-scores. The main limitation of this strategy is its dependence on a representative control sample providing the mean and SD of controls. Control samples in all studies were too small to represent a control population with confidence. However, we checked frequency histograms for compatibility with a normal distribution and think that the excellent differentiation between controls and AD patients, with sensitivity and specificities of Aβ42/40, Aβ42, t-tau, and p-tau^181^ comparable to widely accepted literature values (Blennow et al., 2010) argues in favor of a valid meta-analysis. In addition, the confidence intervals of the AUC decrease with increasing sample size as expected. Further, this is the first meta-analysis on CSF parameters in CAA calculating and reporting standard parameters such as AUC and performing a ROC analysis.

A limitation of all currently available studies is the use of the original or modified Boston criteria on the level of a possible or probable CAA as the gold standard instead of the pathological diagnosis based on a full post-mortem examination or a biopsy. As opposed to some of the previous studies we could include patients with supporting histology. All patients with pathological samples (probable CAA with supporting pathology) in our study suggest that CSF biomarker values in pathologically supported cases are in the same range as the ones without pathological support. This argues in favor of these criteria even in the category “probable” that is mainly based on radiological data. However, in four hospital-based MRI-neuropathological studies the Boston criteria for probable CAA cases showed sensitivities between 42–77% and specificities of 88–100% (Greenberg and Charidimou, 2018). In practice, brain biopsies are rarely conducted, thus the clinician frequently has to rely solely on the MRI and clinical parameters to decide on a future stroke prophylaxis and faces a difficult decision concerning oral anticoagulation or thrombolysis (Banerjee et al., 2017).

The idea to use CSF parameters to diagnose CAA rests on the assumption that assessing the pathological agent itself might improve diagnostic accuracy and could help to make the diagnosis earlier and maybe facilitating opportunities to prevent the progression of the disease in the future (Tanaka et al., 2020). Nevertheless, this study and the quantitative meta-analysis underlines that the differentiation between CAA and AD using CSF parameters analyzed in this study proves to be difficult. One reason could be the overlap in disease mechanisms, both involving Aβ40 and Aβ42 pathology (Noguchi-Shinohara et al., 2017). However, it might be possible to find other CSF biomarkers which are better suited to differentiate between CAA and AD. Current studies of AD biomarkers focus more and more on plasma biomarkers. Most of these studies aim to replace CSF by plasma using the same biomarkers, facilitating sample acquisition. It is unlikely that the discriminatory power of the biomarkers analyzed in this study is higher in plasma than CSF.

If Aβ is not cleared by perivascular drainage from the CSF, it might be deposited as neuritic plaques in the brain parenchyma or as CAA in vessel walls (Greenberg et al., 2020). Since the pathologic noxious agents (Aβ40 and Aβ42) are basically identical in CAA and AD, the diagnostic differentiation of CAA and AD should focus on the secondary consequences of the disease process as reflected in the modified Boston criteria with its emphasis on neuroradiological findings. Furthermore, neuropsychological testing could offer additional help. AD shows a cortical pattern with an emphasis on memory impairment, while CAA patients suffer mainly from subcortical cognitive disabilities, such as attention and executive deficits (Case et al., 2016). Another option might be amyloid-PET but availability, cost, and radiation exposure limit its use (Charidimou et al., 2017). Finally, quantification of diagnostic criteria (e.g., of microbleeds) might improve the modified Boston criteria and might even help estimate the risk of intracerebral hemorrhage (Tsai et al., 2017; Wilson and Werring, 2017).

In conclusion, our study and meta-analysis suggest that Aβ40, Aβ42, t-tau, and p-tau^181^ and the Aβ42/40 ratio are useful in the differentiation between control subjects and CAA patients. However, these markers do not differentiate well enough between AD and CAA patients to be useful in clinical routine. Maybe other diagnostical approaches as mentioned before might be helpful.

## Data Availability Statement

The raw data supporting the conclusions of this article will be made available by the authors upon reasonable request, without undue reservation.

## Ethics Statement

The studies involving human participants were reviewed and approved by the Ethics Committee of the Medical Faculty of the University of Kiel, Lübeck and Tübingen. The ethics committee waived the requirement of written informed consent for participation.

## Author Contributions

NGM, UJ-K, CW, and GK contributed to conception and design of the study and organized data handling. GK performed the statistical analysis. GK and NGM wrote the first draft of the manuscript. UJ-K and FL wrote sections of the manuscript. All authors contributed to data acquisition and analysis and manuscript revision, read, and approved the submitted version.

## Conflict of Interest

The authors declare that the research was conducted in the absence of any commercial or financial relationships that could be construed as a potential conflict of interest.

## Publisher’s Note

All claims expressed in this article are solely those of the authors and do not necessarily represent those of their affiliated organizations, or those of the publisher, the editors and the reviewers. Any product that may be evaluated in this article, or claim that may be made by its manufacturer, is not guaranteed or endorsed by the publisher.

## Footnotes

1
www.cochrane.org

2
www.ClinicalTrials.gov

3
plotdigitizer.sourceforge.net
